# Prognostic role of TOPS in ambulance-transferred neonates in a low-resource setting: a retrospective observational study

**DOI:** 10.1186/s12884-022-05060-9

**Published:** 2022-09-23

**Authors:** Francesco Cavallin, Annaelena Contin, Natércia Alfeu, Belinda Macmillian, Amir Hussein Abubacar Seni, Bonifacio Rodriguez Cebola, Serena Calgaro, Giovanni Putoto, Daniele Trevisanuto

**Affiliations:** 1 Independent Statistician, Solagna, Italy; 2 Doctors With Africa CUAMM Mozambique, Beira, Mozambique; 3 Department of Woman’s and Child’s Health, University Hospital of Padua, Via Giustiniani, 3, 35128 Padua, Italy; 4 Department of Pediatrics, Beira Central Hospital, Beira, Mozambique; 5 Doctors With Africa CUAMM, Padua, Italy

**Keywords:** Neonatal transport, Mortality, TOPS, Low-resource setting

## Abstract

**Background:**

Assessing the severity of transferred neonates at admission can improve resource allocation. This study evaluated the role of TOPS (illness severity score including temperature, oxygen saturation, skin perfusion and blood sugar) in predicting mortality in neonates transferred by ambulance in a low-resource setting.

**Methods:**

The study was conducted at Beira Central Hospital (Mozambique). Infants who were transferred by ambulance to the Neonatal Intensive Care Unit between 16th June and 16th October 2021 were included. The association between TOPS and mortality was investigated with a logistic regression model. Receiver-operating characteristics (ROC) curve was derived for TOPS; area under the ROC curve, sensitivity and specificity were calculated.

**Results:**

In-transport mortality was 2/198 (1.0%) and in-hospital mortality was 75/196 (38.3%). Median gestational age and birthweight were 38 weeks and 2600 g. Main causes of admission were asphyxia (29.3%), prematurity (25.3%) and sepsis (22.7%). Hypothermia and oxygen desaturation at admission were 75.8% and 32.3%. TOPS ≥ 1 was associated with increased mortality risk (odds ratio 7.06. 95% confidence interval 1.90 to 45.82), with 0.97 sensitivity and 0.26 specificity.

**Conclusions:**

The high mortality rate calls for interventions and quality initiative studies to improve the transfer process and the conditions at admission. TOPS can be used to identify neonates at risk of mortality and concentrate efforts of health care providers. Interventions preventing hypothermia and oxygen desaturation should be implemented in pre-transport stabilization and care during transport.

**Supplementary Information:**

The online version contains supplementary material available at 10.1186/s12884-022-05060-9.

## Introduction

According to the last report by the United Nations International Children’s Emergency Fund (UNICEF), over 5 million under-5 children died in 2020, including 2.4 million newborns [1]. While under-5 mortality has been significantly decreasing in the last three decades, a slower reduction rate has been observed in neonatal mortality [1]. Around 1 million of neonatal deaths occurred in sub-Saharan Africa, accounting for 43% of worldwide neonatal mortality [1].

While the centralization of high-risk deliveries is the preferred option, postnatal transport is inevitable when maternal transfer was not performed or possible, or specialized neonatal care was not anticipated before birth [2–5]. Neonatal inter-facility transport is a key aspect of perinatal care that aims to offer the appropriate care to preterm or sickest infants [4].

In low-income countries, many births occur in rural settings or at home, hence several efforts have been focused on promoting institutional births [6]. However, these usually take place in peripheral health centers with basic equipment and health care providers with limited formal training, thus sick babies require transport to a referral facility [6]. While neonatal transport is well established as part of regionalized perinatal care networks in high-income countries [7], transport modalities in low/middle-income countries remain suboptimal and transportation routes are difficult and time-consuming [6, 8–10]. In addition, pre-transport stabilization and care during transport are often inadequate, with potential serious consequences on infant outcome [11].

Assessing the severity at admission to the referral facility can improve resource allocation by health care providers and be used in the evaluation of the improvement in the transport process. TOPS score is an illness severity score for transferred newborns which was created as a simple and useful bedside method including four parameters (temperature, oxygenation, capillary refill time, and blood sugar) [11].

This study aimed to evaluate the role of TOPS in predicting mortality in neonates who were transferred by ambulance in a low-resource setting, with the purpose of identifying opportunities for improvement.

## Methods

### Study design

This retrospective observational study described the neonates who were transferred by ambulance to the Beira Central Hospital (Mozambique). The study was approved by the Comité Interinstitucional de Bioética para Saúde—CIBS/Sofala (prot.005/CIBS/Sofala) and written informed consent was obtained from the parents/caregivers of the newborns.

### Setting

Beira Central Hospital (BCH) is the referral Hospital for the Sofala Province in Mozambique, and accounts for around 6,000 deliveries per year. The health care system in Sofala Province includes 158 health centers (primary level), one district hospital and four rural hospitals (secondary level) and the BCH (referral center) [12].

The Neonatal Intensive Care Unit (NICU) of the BCH is the second largest in the country and admits around 2,200 neonates every year, 56% of them are referred from other health centers or home. NICU staff includes a pediatrician, two general doctors, two residents, 26 nurses and six health workers. The NICU has 14 beds and is equipped with incubators, infant warmers, oxygen, bubble continuous positive airway pressure (CPAP), peristaltic and syringe pumps, phototherapy, and a portable ultrasound machine. Parenteral nutrition, invasive ventilation and therapeutic hypothermia are not available. Kangaroo Mother Care is offered in a dedicated 16-bed room.

Neonates can be referred to BCH by ambulance, public transport (i.e. vans and bus), private vehicles (vans, cars, three-wheel motorbike) or on foot. Ambulances transfer neonates from health centers to BCH, and are equipped with oxygen, self-inflating bag and face mask, thermometer, peripheral venous line, stethoscope, gloves, and delivery-kit. The transport incubator is not available, and neonates are usually transferred in parent’s arms. The transport involves a health care provider (usually a pediatric nurse) when indicated. The service is free-of-charge.

### Patients

Outborn infants who were admitted to the NICU of BCH between 16th June and 16th October 2021 were eligible for inclusion in the study. Infants who were transferred by ambulance were included in the main analysis, while infants transferred by other means of transport and those with incomplete information about transport were excluded.

### Data collection

Data were collected from NICU and transport records, and neonatal medical charts. Two researchers (AC and NA) retrieved the data independently and any inconsistencies were resolved by a third researcher (BM). Retrieved data included patient characteristics at birth, transport data, interventions before and during transport, information at admission and outcome. Gestational age was calculated according to the Last Normal Menstrual Period recall or assessed by using the New Ballard Score [13, 14]. Information on neonatal temperature (< 36.5 °C), oxygenation (SpO2 < 90%), capillary refill time (≥ 3 s) and blood sugar (< 40 mg/dl) at NICU admission were used to calculate the TOPS score (ranging from 0 to 4) in infants with birthweight ≥ 1,000 g and no life-threatening malformations (according to the selection in Mathur et al.) [11].

### Statistical analysis

Numerical data were summarized as median and interquartile range (IQR), and the categorical data as absolute frequency and percentage. Comparisons between groups were performed using Mann–Whitney test (numerical data) and Chi Square test or Fisher’s test (categorical data). The association between TOPS and mortality was investigated with a logistic regression model, adjusting for imbalances at NICU admission. Effect sizes were reported as odds ratio (OR) with 95% confidence interval (CI). Receiver-operating characteristics (ROC) curve was derived for TOPS predicting mortality; area under the ROC curve (AUC), sensitivity, specificity, positive predictive value, and negative predictive value were calculated with their 95% CIs. All tests were 2-sided and a p-value less than 0.05 was considered statistically significant. Statistical analysis was performed using R 4.1 (R Foundation for Statistical Computing, Vienna, Austria) [15].

## Results

### Patient selection

**Fig. 1:**
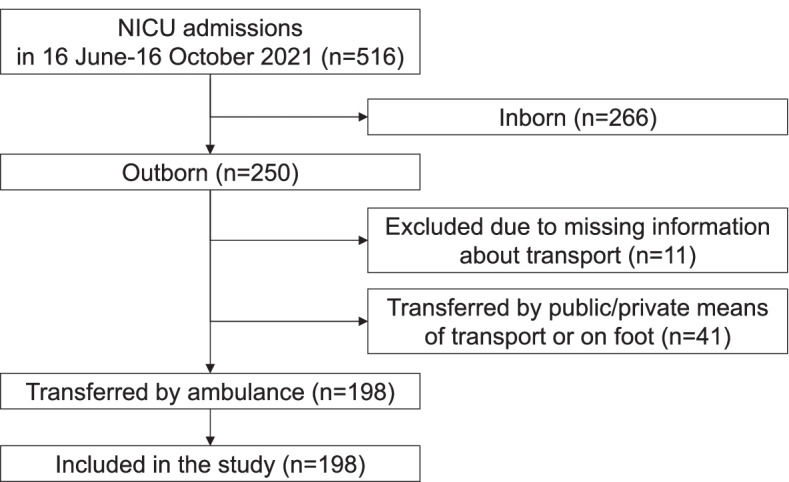
Flowchart of patient inclusion (16 June 2021- 16 October 2021)

Overall, 516 newborns were admitted to the NICU of BCH between 16 June and 16 October 2021 (Fig. 1). Of them, 277 were excluded from the analysis because they were inborn infants (*n* = 266) or outborn infants with missing information about transport (*n* = 11; Supplementary Table 1). Further 41 outborn infants were excluded because were transferred by other means of transport (15 by public/private van, 11 by local three-wheel motorbike, 10 by personal car, four on foot, and one by public local bus) (Supplementary Table 2). The remaining 198 outborn infants who were transferred by ambulance were included in this analysis.

### Characteristics of included patients

Table 1 displays the patient characteristics. The majority was admitted at their first day of life (129/198, 65.2%). The most frequent diagnosis at admission included asphyxia (58/198, 29.3%), prematurity (50/198, 25.3%) and sepsis (45/198, 22.7%). Median distance was 13 km (IQR 7–32).

**Table 1:** Characteristics of outborn infants transferred by ambulance to Beira Central Hospital

Outborn infants transferred with the ambulance	198
Maternal age, years: ^a^	22 (20–29)
Homebirth	21 (10.6)
Mode of delivery:	
Vaginal delivery	195 (98.5)
Caesarean section	3 (1.5)
Males	113 (57.1)
Females	85 (42.9)
Gestational age, weeks ^a^	38 (34–39)
Gestational age:	
< 28 weeks	5 (2.5)
28–31 weeks	26 (13.1)
32–36 weeks	39 (19.7)
37–42 weeks	128 (64.6)
Birth weight, grams: ^ab^	2600 (1778–3000)
Birth weight: ^b^	
< 1000 g	6/194 (3.1)
1000–1499 g	24/194 (12.4)
1500–2499 g	55/194 (28.3)
2500–4000 g	107/194 (55.2)
> 4000 g	2/194 (1.0)
5-min Apgar score:	
0–3	13 (6.6)
4–6	40 (20.2)
7–10	117 (59.1)
Unknown	28 (14.1)
Distance, km ^a^	13 (7–32)
Age at admission, days ^a^	0 (0–3)
Age at admission:	
≤ 24 h	129 (65.2)
> 24 h	69 (34.8)
Weight at admission, grams ^a^	2498 (1668–3025)
Diagnosis at admission:	
Asphyxia	58 (29.3)
Prematurity	50 (25.3)
Sepsis	45 (22.7)
Congenital malformation ^c^	21 (10.6)
Respiratory distress	7 (3.5)
Gastrointestinal diseases ^d^	7 (3.5)
Cutaneous or musculoskeletal diseases ^e^	4 (2)
Metabolic problems ^f^	2 (1)
Convulsions	2 (1)
Poor growth or weight loss	2 (1)

Data expressed as n (%) or ^a^ median (IQR)

Data not available in ^b^4 neonates

^c^Congenital malformations included spina bifida (*n* = 10), abdominal wall defects (*n* = 6), imperforazione anale (*n* = 1), club foot (*n* = 1), hydrocphalus (*n* = 1), neck mass (*n* = 1), sacrococcygeus teratoma (*n* = 1)

^d^Abdominal distension, diarrhea, vomiting

^e^Abscesses, cellulitis, fractures, birth trauma

^f^Hypoglycemia, jaundice

### Interventions before and during transport

Interventions before and during transport are reported in Table 2. Most transports had a written referral letter (170/198, 85.9%), while a pre-transfer phone call to the referral center was less frequent (52/198, 26.3%). Health care providers were present during 160/198 transports (80.8%).

**Table 2:** Interventions before and during transport of outborn infants transferred by ambulance to Beira Central Hospital

Phase	Aspects	Description	Outborn infants transferred by ambulance (*n* = 198)
Before transport	Interventions	Warming	98 (49.5)
		Suctioning airway	70 (35.4)
		Oxygen administration	44 (22.2)
		Face Mask Ventilation	44 (22.2)
		Chest compressions	30 (15.2)
		Adrenaline	3 (1.5)
		Sodium chloride 0.9% infusion	2 (1)
		Dextrose infusion	8 (4)
		Antibiotic therapy	10 (5.1)
		Neonatal prophylaxis (ocular, umbilical)	46 (23.2)
		Breastfeeding	68 (34.3)
	Communication and documentation	Pre-transfer phone call to the referral center	52 (26.3)
		Written referral letter	170 (85.9)
During transport	Interventions	Skin-to-skin contact	40 (20.2)
		Breastfeeding	33 (16.7)
		Oxygen administration	37 (18.7)
		Face Mask Ventilation	4 (2)
		Chest compressions	1 (0.5)
		Adrenaline	0 (0.0)
		Sodium chloride 0.9% infusion	0 (0.0)
		Dextrose infusion	0 (0.0)
		Antibiotic therapy	0 (0.0)
	Health care provider	Nurse	159 (80.3)
		Medical doctor	1 (0.5)
		None	38 (19.2)

Data expressed as n (%)

### TOPS components at admission

Vital signs at admission are shown in Table 3. Regarding the TOPS components, body temperature < 36.5 °C was found in 150/198 neonates (75.8%), oxygen saturation < 90% in 64/198 (32.3%), capillary refill time ≥ 3 s in 22/198 (11.1%) and blood sugar < 40 mg/dl in 14/177 (7.9%). Hypothermia was found in 31/40 (77.5%) neonates receiving skin-to-skin contact and 119/158 (75.3%) not receiving skin-to-skin contact (*p* = 0.94). Among 64 neonates with transcutaneous oxygen saturation < 90% at admission, 18 (28.1%) received supplemental oxygen during transport.

**Table 3:** Vital signs at admission of outborn infants transferred with the ambulance to Beira Central Hospital

Aspect	Variable at admission	Outborn infants transferred by ambulance (*n* = 198)
Clinical parameters	Heart rate:	
	≤ 60 bpm	7 (3.5)
	60–100 bpm	7 (3.5)
	101–180 bpm	182 (92.0)
	> 180 bpm	2 (1.0)
	Respiratory rate:	
	Apnea	8 (4.0)
	< 40 breaths/min	23 (11.6)
	40–60 breaths/min	104 (52.6)
	> 60 breaths/min	63 (31.8)
	Oxygen Saturation:	
	< 80%	27 (13.6)
	80–91%	45 (22.7)
	> 92%	126 (63.6)
	Body temperature:	
	< 32 °C	4 (2.0)
	32–35.9 °C	107 (54.1)
	36–36.4 °C	39 (19.7)
	36.5–37.5 °C	40 (20.2)
	> 37.5 °C	8 (4.0)
TOPS components	Body temperature < 36.5 °C	150 (75.8)
	Oxygen saturation < 90%	64 (32.3)
	Capillary refill time ≥ 3 s	22 (11.1)
	Blood sugar < 40 mg/dl^a^	14/177 (7.9)

Data expressed as n (%)

^a^Data not available in 21 neonates

### Outcome

Two neonates expired at the arrival at the NICU of BCH (one late preterm, asphyxiated infant with 5-min Apgar score of 4 who was transferred after birth with body temperature of 34 °C; one full term, asphyxiated infant with 5-min Apgar score of 3 who was transferred at the 13^th^ day of life with body temperature < 32 °C) and 196 were admitted to the NICU of BCH. Median length of stay was 4 days (IQR 2–8). In-hospital mortality was 75/196 (38.3%).

### Role of TOPS in predicting mortality

**Fig. 2:**
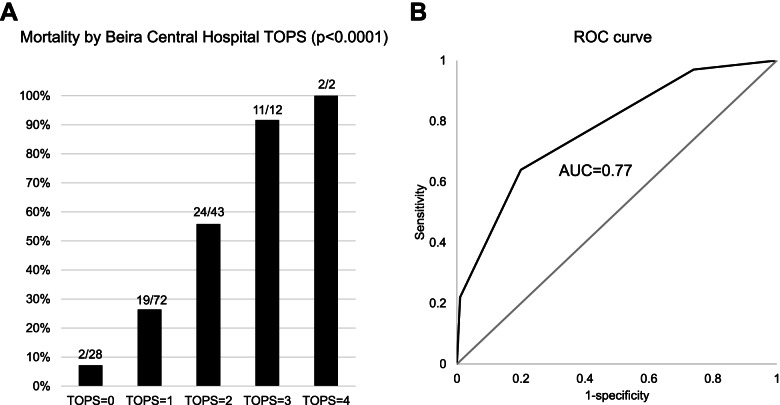
Mortality risk according to TOPS score (**A**) and ROC curve for TOPS predicting mortality (**B**)

Among the 171 outborn infants with birthweight ≥ 1,000 g and no life-threatening malformations (selected according to Mathur et al.) [11], increasing TOPS score was associated with higher mortality risk (*p* < 0.0001; Fig. 2A). The ROC curve suggested sensitivity of 0.97 (95% CI 0.88 to 0.99), specificity of 0.26 (95% CI 0.18 to 0.36), positive predictive value of 0.43 (95% CI 0.35 to 0.52) and negative predictive value of 0.93 (95% CI 0.76 to 0.99) for TOPS ≥ 1; 0.64 (95% CI 0.50 to 0.76), specificity of 0.80 (95% CI 0.71 to 0.87), positive predictive value of 0.65 (95% CI 0.51 to 0.77) and negative predictive value of 0.79 (95% CI 0.70 to 0.87) for TOPS ≥ 2; 0.22 (95% CI 0.13 to 0.35), specificity of 0.99 (95% CI 0.95 to 0.99), positive predictive value of 0.93 (95% CI 0.66 to 0.99) and negative predictive value of 0.69 (95% CI 0.60 to 0.76) for TOPS ≥ 3 (AUC = 0.77, 95% CI 0.70 to 0.84; Fig. 2B). Similar sensitivity and specificity were found when analyzing neonates transferred by ambulance or any other means of transport (Supplementary Table 3).

### Mortality risk factors

In outborn infants (with birthweight ≥ 1,000 g and no life-threatening malformations) who were transferred by ambulance, higher mortality rate was associated with higher TOPS score and its components, lower gestational age, lower birth weight, no pre-transfer phone call, and breastfeeding during transport (Table 4). Multivariable analysis confirmed TOPS ≥ 1 as independent risk factor for mortality (OR 7.06. 95% CI 1.90 to 45.82, *p* = 0.01), adjusting for pre-transfer phone call (OR 0.41, 95% CI 0.17 to 0.92, *p* = 0.03), birth weight (OR 1.00, 95% CI 0.99 to 1.00, *p* = 0.24) and breastfeeding during transport (OR 0.43, 95% CI 0.11 to 1.33, *p* = 0.17). Gestational age was not included in the model due to collinearity with birth weight.

**Table 4:** Mortality risk factors in outborn infants (birthweight ≥ 1,000 g, no life-threatening malformations) transferred by ambulance

Variable	Discharged (*n* = 111)	Dead (*n* = 60)	*p*-value
Body temperature < 36.5 °C	79 (71.2)	52 (86.7)	0.04
Oxygen Saturation < 90%	20 (18.0)	37 (61.7)	< 0.0001
Capillary refill time ≥ 3 s	2 (1.8)	15 (25.0)	< 0.0001
Blood sugar < 40 mg/dl ^b^	5/99 (5.1)	8/58 (13.8)	0.07
TOPS: ^b^			< 0.0001
0	26/99 (26.3)	2/58 (3.4)	
1	53/99 (53.5)	19/58 (32.8)	
2	19/99 (19.2)	24/58 (41.4)	
3	1/99 (1.0)	11/58 (19.0)	
4	0/99 (0.0)	2/58 (3.4)	
Maternal age, years: ^a^	24 (20–30)	22 (20–28)	0.27
Homebirth	9 (8.1)	7 (11.7)	0.63
Males	64 (57.7)	34 (56.7)	0.99
Females	47 (42.3)	26 (43.3)	
Gestational age:			0.03
28–31 weeks	10 (9.0)	14 (23.3)	
32–36 weeks	21 (18.9)	12 (20.0)	
37–42 weeks	80 (72.1)	34 (56.7)	
Birth weight, grams ^a^	2750 (2000–3058)	2380 (1538–2900)	0.02
5-min Apgar score:			0.12
0–3	3 (2.7)	6 (10.0)	
4–6	27 (24.3)	9 (15.0)	
7–10	69 (62.2)	37 (61.7)	
Unknown	12 (10.8)	8 (13.3)	
Distance, km ^a^	13 (7–23)	11 (6–25)	0.23
Age at admission:			0.32
≤ 24 h	69 (62.2)	42 (70.0)	
> 24 h	42 (37.8)	18 (30.0)	
Diagnosis at admission:			0.07
Asphyxia or respiratory distress	37 (33.3)	18 (30.0)	
Prematurity	21 (18.9)	21 (35.0)	
Sepsis	30 (27.1)	15 (25.0)	
Other	23 (20.7)	6 (10.0)	
Before transport:			
Warming	55 (49.5)	31 (51.7)	0.87
Suctioning airway	42 (37.8)	23 (38.3)	0.99
Oxygen administration	29 (26.1)	14 (23.3)	0.72
Face Mask Ventilation	27 (24.3)	15 (25.0)	0.99
Chest compressions	19 (17.1)	9 (15.0)	0.83
Antibiotic therapy	5 (4.5)	5 (8.3)	0.32
Neonatal prophylaxis	30 (27.0)	10 (16.7)	0.14
Breastfeeding	45 (40.5)	16 (26.7)	0.09
Pre-transfer phone call	39 (35.1)	10 (16.7)	0.01
Written referral letter	98 (88.2)	51 (85.0)	0.63
During transport:			
Kangaroo mother care	21 (18.9)	12 (20.0)	0.84
Breastfeeding	21 (18.9)	4 (6.7)	0.04
Oxygen administration	23 (20.7)	10 (16.7)	0.69
Nurse or medical doctor during the transport	91 (82.0)	49 (81.7)	0.99

Data expressed as n (%) or ^a^ median (IQR)

^b^Data not available in 14 neonates. Other diagnoses included respiratory distress (*n* = 6), congenital malformation (*n* = 6), gastrointestinal diseases (*n* = 7), cutaneous or musculoskeletal diseases (*n* = 4), metabolic problems (*n* = 2), convulsions (*n* = 2), poor growth or weight loss (*n* = 2)

## Discussion

Our findings underlined the high rate of hypothermia and desaturation among transferred infants by ambulance in a low-resource setting, and suggested a prognostic role of TOPS.

Despite the promotion of institutional births in low/middle-income countries, the limited resources in peripheral health centers usually force the transfer of sick babies to a referral facility [6]. In agreement with dedicated literature, our data showed that asphyxia, prematurity, and sepsis were the main causes for postnatal transfer, and most babies were transferred during the first day of life [6, 16].

Pre-transport stabilization and care during transport are crucial aspects in the management of these patients [6, 11]. Our data showed suboptimal warming care (half of the babies before transport and none during transport) and high rate of hypothermia at admission to the referral center (75.8%), hence highlighting the need for improvements in thermal management before and during transport. While skin-to-skin contact has been suggested as an effective approach during neonatal transport [17], only one out of five transported babies received skin-to-skin contact. We believe that this finding requires further investigation on application of skin-to-skin contact and/or considerations about alternative warming methods in this setting [18, 19]. Our data also suggested a large underestimation of hypoxia during transport, since most desaturated infants at admission to the referral hospital had not received supplemental oxygen before. Clinical evaluation of cyanosis can be difficult as there is limited agreement between infant color and oxygen saturation, hence a pulse oximeter should be included in the ambulance equipment [20]. These problems occurred despite the frequent presence of a nurse during the transport, which was higher compared to previous studies in low/middle-income countries [9, 21, 22]. Specific training on management of neonates during transport should be offered to health care providers who are involved in this activity. Of note, the referral center often received a written referral letter but was rarely informed before transfer, as previously reported [9, 21]. Our data identified pre-transfer phone call to the referral center as a protective factor for mortality, thus underling the importance of prompt communication between referring and referral centers. We may speculate that both sides can benefit from such communication, as the referring center may receive consultation for pre-transfer stabilization and the referral center may be ready for patient’s arrival.

In our study, we found a high mortality rate in babies who needed postnatal transport, in agreement with literature [9, 23, 24]. Therefore, assessing the severity of transferred babies can improve resource allocation by health care providers at the referral center. Nonetheless, some limitations of the referral center (such as the lack of mechanical ventilation) underline the need for strengthening the local care. Our study evaluated TOPS as simple illness severity score (including temperature, oxygenation, capillary refill time, and blood sugar at admission) which has been suggested as useful predictor of mortality risk in low-middle resource settings [11]. Our data confirmed that TOPS at NICU admission was an independent predictor for mortality in a low-resource setting. We found that at least one derangement in any TOPS component was able to identify almost all neonates at risk of mortality (sensitivity 99%), who would benefit from greater resource allocation. On the other hand, the low specificity (26%) implied a high proportion of babies with low mortality risk who would receive unnecessary attention, hence reducing optimization of resource allocation. Of note, we also reported positive and negative predictive values for TOPS thresholds in the Results section; when considering such findings, the reader should remember that mortality prevalence impacted those statistics. Previous studies suggested a different threshold (derangements of 2 or more components) with better sensitivity/specificity balance (81.6%/77.4% in Mathur et al.; 81.5%/70.6% in Verma et al.; 71.9%/80.8% in Begum et al.) which may result in improved resource allocation but higher mortality [11, 23, 25]. In our data, derangements of 2 or more components provided comparable specificity but lower sensitivity, due to higher mortality among neonates with only one deranged component. Such discrepancy may be due to the different setting (sub-Saharan Africa vs. India), the different transferring system (referring center, transport service and referral center) and means of transport (by ambulance vs. ambulance and any other means). We replicated our analysis in neonates transferred by ambulance or other means of transport (Supplementary Table 3), and found similar results (comparable specificity but lower sensibility with respect to previous studies), hence we may speculate that different setting and transferring system may explain the discrepancy in sensitivity. Of note, the primary analysis focused on transport by ambulance because being transferred by ambulance or other means implied different subpopulations (for example, neonates transported by other means were older, less sick and cared for by unspecialized caregivers), as confirmed in Supplementary Table 2. In addition, there was a high heterogeneity among the other means of transport, including public van, private van, local three-wheel motorbike, personal car, public bus, or on foot. Further investigations in larger samples and different settings may provide more information on the optimal alert signal to stratify risk of mortality in transferred neonates.

Our study adds information on the prognostic role of TOPS in neonates transferred by ambulance in a low-resource setting, and offers useful insights about the care before and during the transport. This study has some limitations that should be considered. First, this is a single-center study hence the generalizability of the findings should be limited to similar settings. Second, the retrospective design precludes any causal relationship. Third, TOPS at referring centers and data on transport time were not available.

## Conclusions

The high mortality rate calls for interventions and quality initiative studies to improve the transfer process and the conditions at admission. TOPS can be used to identify neonates at risk of mortality and concentrate efforts of health care providers. Interventions preventing hypothermia and oxygen desaturation should be implemented in pre-transport stabilization and care during transport.

## Supplementary Information

**Additional file 1:** **Supplementary Table 1.** Characteristics of outborn infants who were excluded due to incomplete information about transport. **Supplementary Table 2.** Information about outborn infants admitted to Beira Central Hospital according to means of transport. **Supplementary Table 3.** Sensitivity and specificity of TOPS score for prediction of mortality in 210 outborn infants with birthweight ≥1,000 grams and no life-threatening malformations (who were admitted to Beira Central Hospital between 16 June and 16 October 2021) transferred by ambulance or other means of transport.

## Data Availability

The datasets used and/or analysed during the current study are available from the corresponding author on reasonable request.
