# Supplementary material for: Prognostic role of TOPS in ambulance-transferred neonates in a low-resource setting: a retrospective observational study
Source: BMC Pregnancy Childbirth. 2022 Sep 23;22:726. doi: 10.1186/s12884-022-05060-9 (PMC9503278; doi:10.1186/s12884-022-05060-9)
Supplement: Supplementary file 1 — Additional file 1: Supplementary Table 1. Characteristics of outborn infants who were excluded due to incomplete information about transport. Supplementary Table 2. Information about outborn infants admitted to Beira Central Hospital according to means of transport. Supplementary Table 3. Sensitivity and specificity of TOPS score for prediction of mortality in 210 outborn infants with birthweight ≥1,000 grams and no life-threatening malformations (who were admitted to Beira Central Hospital between 16 June and 16 October 2021) transferred by ambulance or other means of transport. [file 12884_2022_5060_MOESM1_ESM.docx]

**SUPPLEMENTARY TABLES**

**Supplementary Table 1. Characteristics of outborn infants who were excluded due to incomplete information about transport**

| N infants | 11 |
| --- | --- |
| Sex  Male  Female | 7 (63.6%)  4 (36.4%) |
| Provenience:  Beira City  Sofala Province | 6 (54.5%)  5 (45.5%) |
| Main diagnosis at admission:  Sepsis  Prematurity  Neonatal asphyxia  Congenital malformation  Jaundice | 4 (36.4%)  3 (27.3%)  2 (18.2%)  1 (9.1%)  1 (9.1%) |
| Outcome:  Discharged  Dead | 8 (72.7%)  3 (27.3%) |

Data expressed as n (%).

**Supplementary Table 2. Information about outborn infants admitted to Beira Central Hospital according to means of transport**

| Aspect | Variable | Outborn infants transferred by ambulance (n=198) | Outborn infants transferred by means of transport (n=41) | p-value |
| --- | --- | --- | --- | --- |
| Characteristics | Maternal age, years: ^a^ | 22 (20-29) | 23 (19.26) | 0.39 |
|  | Homebirth: ^b^ | 21 (10.6) | 3/40 (7.5) | 0.76 |
|  | Mode of delivery:  Vaginal delivery  Caesarean section | 195 (98.5)  3 (1.5) | 32/40 (80.0)  8/40 (20.0) | <0.0001 |
|  | Males  Females | 113 (57.1)  85 (42.9) | 24 (58.5)  17 (41.5) | 0.99 |
|  | Gestational age, weeks ^a^ | 38 (34-39) | 38 (38-38) | 0.25 |
|  | Gestational age:  <28 weeks  28-31 weeks  32-36 weeks  37-42 weeks | 5 (2.5)  26 (13.1)  39 (19.7)  128 (64.6) | 0 (0.0)  1 (2.4)  7 (17.1)  33 (80.5) | 0.14 |
|  | Birth weight, grams: ^ac^ | 2600 (1778-3000) | 2900 (2500-3150) | 0.01 |
|  | Birth weight: ^c^  <1000 grams  1000-1499 grams  1500-2499 grams  2500-4000 grams  > 4000 grams | 6/194 (3.1)  24/194 (12.4)  55/194 (28.3)  107/194 (55.2)  2/194 (1.0) | 0/39 (0.0)  1/39 (2.6)  8/39 (20.5)  29/39 (74.3)  1/39 (2.6) | 0.09 |
|  | 5-minute Apgar score:  0-3  4-6  7-10  Unknown | 13 (6.6)  40 (20.2)  117 (59.1)  28 (14.1) | 1 (2.4)  4 (9.8)  29 (70.7)  7 (17.1) | 0.30 |
| Transport | Antibiotic therapy before transport | 10 (5.1) | 1 (2.4) | 0.70 |
|  | Breastfeeding before transport | 68 (34.3) | 33 (80.5) | <0.0001 |
|  | Distance, km ^a^ | 13 (7-32) | 7 (4-13) | 0.01 |
|  | Written referral letter | 170 (85.9) | 9 (22.0) | <0.0001 |
|  | Skin-to-skin contact during transport | 40 (20.2) | 3 (7.3) | 0.08 |
|  | Breastfeeding during transport | 33 (16.7) | 8 (19.5) | 0.83 |
| Admission | Age at admission:  ≤24 h  >24 h | 129 (65.2)  69 (34.8) | 3 (7.3)  38 (92.7) | <0.0001 |
|  | Weight at admission, grams ^a^ | 2498 (1668-3025) | 2650 (2115-3185) | 0.04 |
|  | Diagnosis at admission:  Asphyxia  Prematurity  Sepsis  Congenital malformation  Respiratory distress  Gastrointestinal diseases  Cutaneous or musculoskeletal diseases  Metabolic problems  Convulsions  Poor growth or weight loss | 58 (29.3)  50 (25.3)  45 (22.7)  21 (10.6)  7 (3.5)  7 (3.5)  4 (2)  2 (1)  2 (1)  2 (1) | 0 (0.0)  2 (4.8)  19 (46.3)  5 (12.2)  2 (4.8)  1 (2.4)  7 (17.1)  3 (7.3)  2 (4.8)  0 (0.0) | <0.0001 |
|  | Body temperature <36.5°C | 150 (75.8) | 22 (53.6) | 0.007 |
|  | Oxygen saturation <90% | 64 (32.3) | 6 (14.6) | 0.04 |
|  | Capillary refill time ≥3 sec | 22 (11.1) | 0 (0.0) | 0.05 |
|  | Blood sugar <40 mg/dl ^d^ | 14/177 (7.9) | 0/37 (0.0) | 0.16 |
| Outcome | Mortality | 77 (38.9) | 5 (12.2) | 0.002 |

Data expressed as n (%) or ^a^ median (IQR). Data not available in ^b^1, ^c^6 and ^d^25 neonates.

**Supplementary Table 3. Sensitivity and specificity of TOPS score for prediction of mortality in 210 outborn infants with birthweight ≥1,000 grams and no life-threatening malformations (who were admitted to Beira Central Hospital between 16 June and 16 October 2021) transferred by ambulance or other means of transport**

|  | Sensitivity (95% confidence interval) | Specificity (95% confidence interval) | Positive predictive value (95% confidence interval) | Negative predictive value (95% confidence interval) |
| --- | --- | --- | --- | --- |
| TOPS≥1 | 0.97 (0.89 to 0.99) | 0.32 (0.24 to 0.41) | 0.40 (0.32 to 0.49) | 0.95 (0.85 to 0.99) |
| TOPS≥2 | 0.66 (0.62 to 0.77) | 0.84 (0.76 to 0.90) | 0.66 (0.52 to 0.77) | 0.84 (.076 to 0.90) |
| TOPS≥3 | 0.21 (0.12 to 0.34) | 0.99 (0.96 to 0.99) | 0.93 (0.66 to 0.99) | 0.73 (0.66 to 0.79) |
